# Prognostic Value of Postneoadjuvant Chemotherapy Neutrophil-to-Lymphocyte Ratio in Patients undergoing Radical Cystectomy

**DOI:** 10.3390/jcm13071953

**Published:** 2024-03-28

**Authors:** Krystian Kaczmarek, Bartosz Małkiewicz, Adam Gurwin, Wiktor Mateusz Krawczyk, Karolina Skonieczna-Żydecka, Artur Lemiński

**Affiliations:** 1 Department of General and Oncological Urology, Independent Provincial Public Integrated Hospital, Arkońska 4, 71-455 Szczecin, Poland; 2 Department of Minimally Invasive and Robotic Urology, University Center of Excellence in Urology, Wroclaw Medical University, Borowska 213, 50-556 Wrocław, Poland; bartosz.malkiewicz@umed.wroc.pl (B.M.);; 3 Department of Urology and Urological Oncology, Pomeranian Medical University, Powstańców Wielkopolskich 72, 70-111 Szczecin, Poland; wiktor.mateusz.krawczyk@gmail.com; 4 Department of Biochemical Sciences, Pomeranian Medical University, Władysława Broniewskiego 24, 71-460 Szczecin, Poland; karolina.skonieczna-zydecka@pum.edu.pl

**Keywords:** bladder cancer, neutrophil-to-lymphocyte ratio, neoadjuvant therapy, radical cystectomy

## Abstract

**Background:** Neutrophil-to-lymphocyte ratio (NLR), a widely assessed biomarker in most common diseases, is typically evaluated before treatment initiation. However, data on NLR in the post-treatment setting is limited. Therefore, we assessed the NLR calculated after neoadjuvant chemotherapy (NAC) initiation in patients with bladder cancer (BC). We hypothesised that changes in blood cells after NAC could be a marker of tumour response and long-term survival. **Materials and Methods:** Our study included 214 patients who underwent NAC followed by radical cystectomy (RC) in two urological departments, wherein post-NAC NLR was used to categorize patients into the low (NLR ≤ 1.75) and high (NLR > 1.75) groups. **Results:** Logistic regression analysis indicated that a post-NAC NLR ≥ 1.75 is a good biomarker for pathologic response (odds ratio (OR), 0.045; *p* <0.001), emphasizing its ability to predict patient survival. The HRs for overall survival and cancer-specific survival were 2.387 (*p* = 0.048) and 2.342 (*p* < 0.001), respectively. **Conclusions:** We believe that post-NAC NLR can be used for patient stratification after NAC. Consequently, the post-NAC NLR may serve as a guide for the decision-making process regarding RC versus bladder-preserving strategies.

## 1. Introduction

Over the past few decades, neoadjuvant chemotherapy (NAC) has become the mainstay treatment for bladder cancer (BC) before the use of radical cystectomy (RC) or chemoradiation (CRT) [1]. NAC has been reported to significantly improve patient prognosis, increasing the 5-year and 10-year overall survival (OS) by 8% and 6%, respectively [2,3]. However, the histopathological response remains to be the surrogate marker of long-term oncological benefits. In NAC, the pathologic response in RC specimens is mostly defined as achieving a tumour stage of ypT0ypN0 or ≤ypT1ypN0, which is observed in approximately 40–50% and 20–30% of patients, respectively [4,5]. In terms of CRT, an advanced bladder cancer (ABC) meta-analysis including 2688 participants showed that the survival benefit of platinum-based NAC persisted irrespective of further treatment, including RC, radiotherapy, or CRT. The results of this meta-analysis are consistent with a previous randomised controlled trial (RCT) [3,5,6]. Regardless of treatment after NAC administration, the response mainly depends on the chemotherapy regimen offered. Now, the dose-dense methotrexate, vinblastine, doxorubicin, and cisplatin (ddMVAC) regimen is considered the most effective [4]. The results of the GETUG-AFU V05 VESPER trial showed that three-year progression-free survival was improved in patients who received ddMVAC before RC over the patients who were exposed to the gemcitabine and cisplatin (GC) regimen [7]. Despite the role of cisplatin-based chemotherapy in the NAC setting being well established, the current landscape of perioperative treatment for MIBC is still developing. Immunotherapy with checkpoint inhibitors as a monotherapy, or in different combinations with or without chemotherapy, is being tested in phase II and III trials. The initial results these of trials are encouraging. For instance, in a single arm of the PURE-01 trial, 42% of patients who received three cycles of pembrolizumab before RC achieved a complete response [8]. Additionally, tumour histopathology plays an important role in chemosensitivity, wherein the most favourable outcomes of NAC have been observed in pure urothelial carcinoma and small cell neuroendocrine variants of BC [9]. Other prognostic factors have also been extensively investigated in BC prognostication. Currently, out of the inflammatory parameters, a systemic immune–inflammation index (SII) is considered as a promising predictor of BC outcomes. However, the results of studies regarding the predictive role of the SII in BC are still widely varied. Moreover, SII was poorly investigated in the NAC setting [10]. Whereas the results for other inflammatory ratios are more consistent, particularly the neutrophil-to-lymphocyte ratio (NLR), which has emerged as a promising tool for prognostication. Neutrophils play different promoting roles in the tumour microenvironment, either by their status or the presence of TGF-β. Moreover, they contribute to the invasion, proliferation, metastasis, and even escape of cancer cells from the immune surveillance system. In contrast, lymphocytes play a crucial role in tumour defence by inhibiting tumour cell migration and proliferation. However, they have been implicated to induce tumour cells [11]. Furthermore, poor survival in cancer has been observed with the diagnosis of leucopenia, which acts as a marker of immune ineffectiveness [12,13]. In recent studies, the link between NLR and BC prognosis has been widely studied in pretreatment settings. Notably, the NLR obtained before a transurethral resection of bladder tumour (TURBT) was reported to predict disease recurrence, as well as survival outcomes in patients who underwent upfront RC [14,15]. These results were confirmed in a meta-analysis by Lucca et al. [12], in which advanced tumour stage and lymph node metastasis have been associated with worse prognosis in patients with a high preoperative NLR. Regarding the multidisciplinary management of muscle-invasive bladder cancer (MIBC), the NLR before the first NAC cycle has been thought to predict response to NAC, cancer-specific survival (CSS), and overall survival (OS). Thus, pre-NAC NLR is considered a simple and inexpensive risk biomarker for BC. However, administration of a complete course of NAC may significantly change the levels of blood cells, resulting in different NLR values post-NAC. Since blood cells are assessed after exposure to treatment, NLR after treatment may better reflect the efficacy of the offered regimens, as well as provide more accurate prognostications. Therefore, we assessed post-NAC NLR in patients undergoing RC, focusing on the correlations between post-NAC NLR and pathologic response and survival outcomes. Our goal was to determine whether post-NAC NLR showed similar predictive ability as that reported in pre-NAC NLR from previous studies.

## 2. Materials and Methods

### 2.1. Ethical Approval

This retrospective study was exempt from further review by the Institutional Review Board (Bioethical Committee) of the Pomeranian Medical University, Szczecin, Poland, and was conducted according to the regulations of the Declaration of Helsinki. Written informed consent was routinely obtained from all patients involved, specifically for the use of anonymised treatment data collected during hospitalisation. 

### 2.2. Inclusion and Exclusion Criteria 

Consecutive patients who underwent NAC followed by RC and PLND for MIBC between 2015 and 2021 at two university centres, the Department of Urological Oncology of the Pomeranian Medical University, Szczecin and the University Center of Excellence in Urology of Wroclaw Medical University, Poland were included in the present study. Patients with the following characteristics were excluded: received fewer than three NAC cycles, confirmed metastatic disease on preoperative computed tomography, underwent cystectomy for palliative indications (e.g., pain, haematuria), prior pelvic radiotherapy, underwent partial bladder resections, and non-urothelial pathology. After the exclusion of 99 patients, only 214 patients were ultimately included for statistical analyses. The criteria proposed by Galsky et al. in 2011 for cisplatin-based chemotherapy in metastatic urothelial carcinoma has been widely utilised in both departments [16]. Two regimens of cisplatin-based chemotherapy were administered: gemcitabine and cisplatin (GC) and ddMVAC. Patients who qualified for the GC regimen received gemcitabine 1000 mg/m^2^ on days 1, 8, and 15 plus cisplatin 70 mg/m^2^ on day 2. These cycles were repeated every 28 days, with a maximum of six cycles of treatment [17]. Meanwhile, those who qualified for the ddMVAC regimen received methotrexate 30 mg/m^2^ on day 1 and vinblastine 3 mg/m^2^, doxorubicin 30 mg/m^2^, and cisplatin 70 mg/m^2^ on day 2. These cycles were repeated every 14 days, with a maximum of four cycles of treatment [18]. Both regimens, which have comparable efficacies, were selected by a multidisciplinary team, with the GC regimen as the preferred management for patients with more comorbidities [17]. It was shown in the GETUG-AFU V05 VESPER trial that the GC regimen has lower toxicity in terms of asthenia and gastrointestinal side effects [19]. If patients did not meet all the criteria of Galsky et al., other regimens within the NAC framework were considered [16]. Carboplatin-based chemotherapy was suggested for patients with an Eastern Cooperative Oncology Group Performance Status (ECOG PS) ≥ 2 and a glomerular filtration rate (GFR) of 30–60 mL/min. Nevertheless, in patients with adequate bone marrow reserves, taxane-based chemotherapy was considered for those with a GFR < 60 mL/min or ECOG PS < 2 [20]. During the analysis period, immuno-oncology therapy was not administered in a neoadjuvant setting. RC with bilateral lymphadenectomy was performed within 4–8 weeks of the final NAC cycle. Pathologic complete response (pCR) to NAC was defined as achieving the ypT0pN0 stage, while pathologic partial response (pPR) was described as downstaging to the <ypT2N0 stage. Patients with persistent MIBC or those who experienced disease progression after NAC exposure were classified as non-responders. 

### 2.3. Measures and Outcomes 

**Figure 1:**
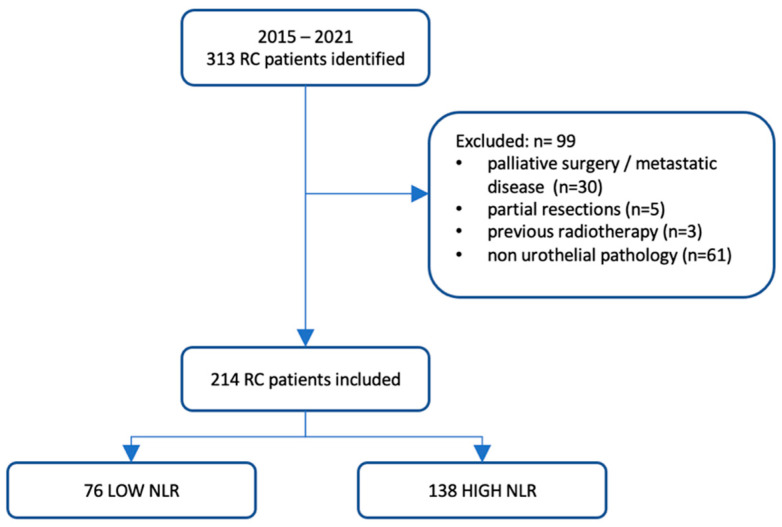
Flowchart of the study. NLR: neutrophil-to-lymphocyte ratio; RC: radical cystectomy.

NLR was calculated 1 day before RC by dividing the absolute neutrophil count by the lymphocyte count as a part of the complete blood cell count. Patients were then divided into two groups according to the NLR. The first group comprised patients with a low NLR, whereas the control group comprised patients with a high NLR (Figure 1). Finally, both groups were compared in terms of pathological response to NAC, OS, and CSS. OS was defined as the period from the date of RC to either the date of death or the last recorded follow-up, with no restriction on the cause of death. CSS was defined as the period from the date of RC to death from BC.

### 2.4. Statistical Analysis

Data consistency was reviewed by two authors (K.K. and A.L.). Normally distributed data were presented as means with standard deviations, whereas skewed data were presented as medians with interquartile ranges. Receiver operating characteristic (ROC) curve analysis was used to investigate whether post-NAC NLR could distinguish between responders and non-responders. The NLR value with the best accuracy (the highest sensitivity [SN] and specificity [SP]) was selected as the post-NAC NLR cutoff. Differences between the high and low post-NAC NLR groups were determined using independent *t*-tests for parametric variables and chi-square tests for nonparametric variables. Kaplan–Meier survival estimates, along with univariate Cox analysis, were also employed to show OS and CSS across time, utilizing the log-rank test to compare survival curves. Additionally, multivariate Cox proportional hazard models were used to assess the impact of preoperative prognostic factors, including age at surgery, sex, obesity (body mass index ≥ 30 kg/m^2^), severity of comorbidities based on the American Society of Anesthesiologists (ASA) score, smoking status (never, former, and current smoker), clinical T stage, post-NAC NLR, and NAC regimen. Schoenfeld residuals were also utilised to examine the independence between the residuals and time, which is essential for verifying the proportional hazard assumption of the concluding multivariable models. Multivariate Cox regression results were presented as hazard ratios (HRs) with 95% confidence intervals (CIs). Additionally, univariate and multivariate analyses were performed for predictors of response (<ypT2N0) to NAC. The threshold for statistical significance was established at 0.05, and all *p*-values were two-sided. Analytical procedures were performed using the Statistica software (version 13.5) (StatSoft, Inc., Tulsa, OK, USA), R (version 4.2.2), and RStudio (version 2022.12.0) with the R packages Survival, Survminer, and Drylr.

## 3. Results

In total, 214 patients underwent the final analysis, which predominantly included males (77.10%) with an overall average age of 66.16 years (standard deviation, +/− 6.94 years). Most patients were preoperatively treated using the ddMVAC regimen (57.94%), and pCR and pPR were achieved in 40 (18.69%) and 75 (35.04%) patients, respectively. Notably, while no significant differences in downstaging rates were observed between the two departments, a tendency towards lower response rates were observed in Wroclaw compared to Szczecin. Specifically, pCR was observed in 19.72% of patients in Szczecin, whereas 16.67% of patients in Wroclaw achieved pCR (*p* = 0.588). A similar non-significant difference was observed in the pPR of both departments (37.32% and 30.56%, respectively; *p* = 0.326). Furthermore, the two departments differed significantly in their preferred cytotoxic regimens. In Szczecin, 102 (71.83%) patients were exposed to the ddMVAC regimen, while in Wroclaw, only 22 (30.56%) patients were exposed to the ddMVAC regimen (*p* < 0.001).

### 3.1. ROC Analysis

**Figure 2:**
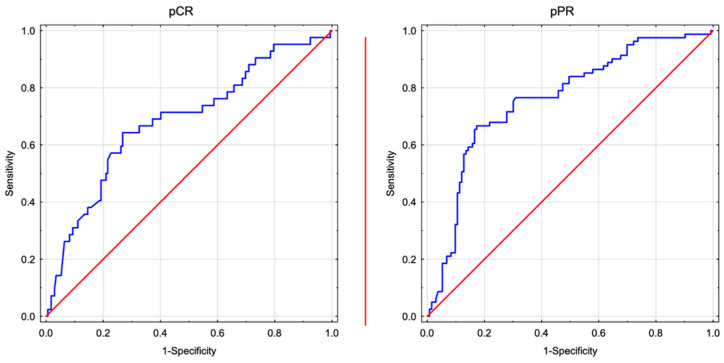
ROC curve analysis for the optimal cutoff NLR value to discriminate patients based on NAC response. Take-home message: post-NAC NLR is associated with local response. pCR: pathological complete response; pPR: pathological partial response.

The median neutrophil and lymphocyte levels were 4.81 × 10^9^/L and 1.80 × 10^9^/L, respectively. ROC analysis identified the optimal NLR cutoff value of 1.75 for predicting downstaging to <ypT2N0. The area under the curve (AUC), SN, and SP were 0.798 (95% CI, 0.736–0.861), 72.00% (95% CI, 60.44–81.76%), and 83.45% (95% CI, 76.21–89.21%), respectively. The optimal NLR cutoff value for predicting pCR was 1.70, with the corresponding AUC, SN, and SP values of 0.711 (95% CI, 0.620–0.802), 67.50% (95% CI, 50.87–81.43%), and 73.56% (95% CI, 66.36–79.95%), respectively (Figure 2). Based on the cutoff value of 1.75, 76 (35.51%) and 138 patients (64.49%) were categorised into the low and high NLR groups, respectively. 

### 3.2. Comparison of Analysed Groups

Patients in the lower NLR group had a favourable pathologic stage distribution, with a higher proportion of ypT0N0 (35.52% vs. 9.42%, *p* < 0.001) and a lower incidence of extravesical disease (23.68% vs. 58.55%, *p* < 0.001). Table 1 compares the clinical–pathological characteristics between the two groups. Notably, no significant differences in any of the analysed preoperative variables were observed between the two groups. 

### 3.3. High post-NAC NLR Is Associated with a Worse Local Response

A multivariate analysis revealed NLR as an independent factor in predicting downstaging to <ypT2N0 in patients exposed to NAC, with a corresponding OR of 0.045 (95% CI, 0.017–0.119; *p* < 0.001; Table 2). Additionally, univariate and multivariate analyses indicated that a high post-NAC NLR is significantly associated with pCR, with corresponding OR values of 0.189 (95% CI, 0.090–0.395; *p* < 0.001) and 0.161 (95% CI, 0.066–0.394; Table S1).

### 3.4. High post-NAC NLR Is Associated with Worse Overall Survival 

**Figure 3:**
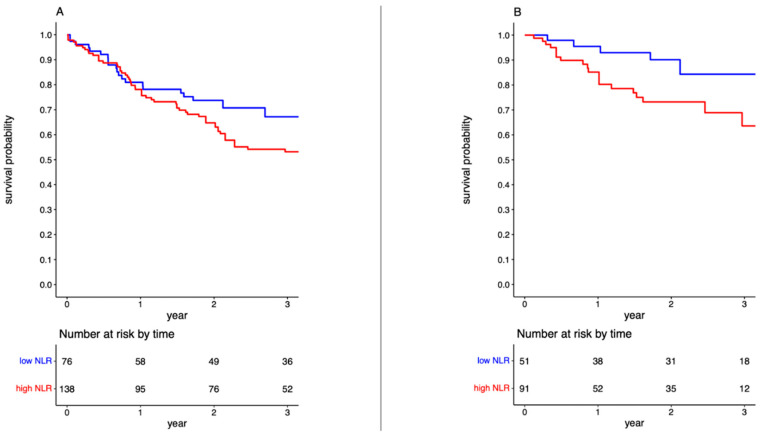
Kaplan–Meier Curve comparing overall survival rates between patients with NLR ≤ 1.75 (blue) and those with NLR > 1.75 (red) (**A**); Kaplan–Meier Curve comparing cancer-specific survival rates between patients with NLR ≤ 1.75 (blue) and those with NLR > 1.75 (red) (**B**). Take-home message: post-NAC NLR is associated with survival outcomes.

The median follow-up duration was 28 months. Of the 215 patients, 118 died at a median time of 29 months. The cause of death was documented in 142 patients, with 28 (19.71%) deaths attributed to BC at a median time of 15 months. The overall 3-year OS for the entire cohort was 58.29% (95% CI, 51.22–65.36%). Patients with a low NLR had a significantly better OS than those with a high NLR (*p* = 0.001), showing 3-year OS rates of 67.18% (95% CI, 56.05–78.32%) and 53.16% (95% CI, 44.17–62.15%), respectively (Figure 3). This association is further confirmed with a corresponding HR of 2.342 (95% CI, 1.511–3.630; *p* < 0.001). Multivariate analysis identified the following variables as independent prognostic factors for OS (HR, 1.686; 95% CI, 1.049–2.711; *p* = 0.031): smoking status and clinical T stage (Table 3). 

### 3.5. High post-NAC NLR Is Associated with Worse Cancer-Specific Survival 

The overall 3-year CSS for the entire cohort was 72.81% (95% CI, 63.06–82.56%). Compared to OS, the low NLR group yielded significantly better CSS rates than that in the high NLR group (*p* = 0.036), showing 3-year CSS rates of 77.30% (95% CI, 60.33–94.26%) and 63.61% (95% CI, 47.95–79.27%), respectively (Figure 3). Although univariate analysis identified NLR as a significant factor for CSS prediction (HR, 2.387; 95% CI, 1.008–5.653; *p* = 0.048), multivariate analysis did not confirm this result (HR, 2.069; 95% CI, 0.789–5.427; *p* = 0.140) (Table 3).

## 4. Discussion

Our study assessed the prognostic utility of the NLR in 214 consecutive patients receiving neoadjuvant regimens before RC. The present study showed that high post-NAC NLR was an independent prognostic factor for long-term outcomes and significantly correlated with poor pathological response. Although the prognostic role of NLR in BC treatment has been established in previous studies, the majority of these reports focused on RC alone or used pre-NLR before the initial NAC cycle. Consequently, the estimated NLR cutoff in our study, which was 1.75, differed from those in other studies, reporting a typical pre-NAC NLR cutoff of 3 [21,22,23]. The prognostic threshold of the estimated NLR in our study was also lower than the calculated prognostication in non-MIBC cases [14]. This phenomenon may be attributed to blood component alterations after NAC administration. Yamada et al. observed a post-therapy NLR decrease driven mainly by significant neutrophil count reduction, suggesting that chemoresistant tumours may exhibit a high post-NAC NLR [24]. However, data regarding changes in NLR before and after NAC administration in BC are limited and imprecise. Another study by Herzberg et al. analysed the early changes in NLR after RC, stating that both preoperative and postoperative high NLR values were significant predictors of poor survival. However, in their study, only 41% of patients received NAC, and the authors did not provide a separate analysis of patients who were and were not pretreated with NAC. As such, the NLR threshold for predicting the survival outcomes of patients exposed to NAC was not estimated. Currently, multidisciplinary treatment has become the standard of care for patients with MIBC [25]. In a study by Kasier et al. involving patients pretreated with NAC before RC, NLR values obtained prior to and after NAC administration were compared. The median NLR during in their study (1.8) was comparable to the cutoff point in our analysis (1.8). Similar to our analysis, they also reported that a sustained high post-NAC NLR was significantly associated with poor outcomes. However, only 43 of their 376 patients experienced a post-NAC NLR decrease. This may be caused by differences in the inclusion criteria, wherein all patients with stage cT2-4aN0M0 disease were treated with at least one cycle of NAC. Therefore, a significant proportion of the patients in that study did not receive adequate NAC exposure, leading to absent findings of NLR decrease in most patients [26]. By limiting our study to patients with at least three NAC cycles, we believe that our NLR threshold more accurately identifies early responses to NAC. 

Regarding pathological response, our cutoff value was higher than those reported in previous pre-NAC settings. In the present study, the SN, SP, and AUC values for NLR > 1.75 were 72.00%, 83.45%, and 0.798, respectively. In studies where NLR was analysed in patients without NAC pretreatment, SN reached approximately 50%, while SP rarely exceeded 80% [14]. Accordingly, our study showed that the number of patients with low NLR who responded to NAC was markedly higher than that in previous reports [23]. We found that 69.74% of patients with an NLR ≤ 1.75 showed a pathologic response to NAC; however, only 15.94% of those with an NLR > 1.75 were downstaged to ypT2N0 disease (*p* < 0.001). Furthermore, multivariate analysis revealed that an NLR > 1.75 predicted a lower pathological response rate to NAC. In a multicentre study by Black et al., NLR was determined as a poor indicator to distinguish between responders and non-responders. In that study, patients were classified into low and high NLR groups based on their pre-NAC NLR, showing that patients with an NLR > 3 were more likely to have residual MIBC than those with an NLR ≤ 3 (70.8% vs. 58.3%). However, this result was almost statistically insignificant (*p*-value = 0.049) [23]. Both these studies suggest that post-NAC NLR can provide more accurate predictions of systemic therapy response, which must be validated with RCTs. In such trials, patients should be randomly assigned to the NAC or non-NAC group, and the NLR should be assessed before the first and after the last NAC cycle. In our study, the lack of pre-NAC laboratory data precluded its analysis, further emphasizing the lack of data in the literature. Although, direct comparisons between pre-NAC and post-NAC NLR have been reported for other cancers, Sanna et al. analysed the accuracy of the pre-NAC and post-NAC NLR in predicting the outcomes of patients with advanced ovarian cancer. They found that the NLR after three NAC cycles yielded superior diagnostic accuracy compared to baseline NLR, with an SN and SP of 79% and 100%, respectively. Moreover, the optimal post-NAC NLR cutoff in their study was 1.58, which was close to our cutoff value [27]. Another study by Yamada et al. emphasised that the post-NAC NLR was a better prognostic factor for OS in patients with oesophageal squamous cell carcinoma [24]. Similarly, high post-chemotherapy NLR was associated with worse tumour response, greater risk of death, and favourable predictive value in patients with metastatic or advanced lung adenocarcinoma [28]. Considering all these findings, we believe that a high post-chemotherapy NLR may imply resistance to treatment and poorer prognosis, and that post-NAC NLR better reflects immunological changes in the tumour microenvironment after NAC exposure.

This study suggests the utility of post-NAC NLR in predicting patient survival. Although multivariate analysis confirmed this for OS, only univariate analysis confirmed this for CSS. In previous pre-NAC studies, NLR was an independent predictor of CSS in both univariate and multivariate analyses [29,30,31,32]. Particularly, one of the largest studies in this field demonstrated that patients with a high pre-NAC NLR had a 1.21-fold higher risk of death from urothelial BC (95% CI, 1.07–1.37) [12]. This discrepancy likely results from the difference in sample size in our study. Since the data regarding the cause of death were only available for some patients, a smaller subset underwent analysis of pre-NAC NLR for CSS.

Interpreting our results, it should be noted that the NLR level might depend on comorbidities. Corriere et al. showed that NLR is a strong predictor of the presence and the number of carotid atherosclerotic plaques [33]. Whereas Tamaki et al. proved that a high NLR is independently associated with cardiac death [34]. Moreover, Wan et al. found associations of NLR level with diabetic complications including cardiovascular and diabetic kidney disease [35]. In our study, we did not assess the correlation between the post-NAC NLR and comorbidity burden. Hence, we are unable to determine whether comorbidities have any effect on blood cell levels after NAC. Nevertheless, we are aware that such a correlation might exist. An analysis of patient comorbidities along with the post-NAC NLR might better predict long-term survival outcomes and enable more precise patient stratification for further treatment. 

Despite the interesting findings of our analysis, the present study has several limitations. First, due to the retrospective nature of this study and its moderate sample size, we were unable to control for all confounding variables, including ECOG PS, Charlson Comorbidity Index, socioeconomic status, and administration of adjuvant chemotherapy. These variables may have a meaningful influence on long-term patient prognosis. Second, comparisons between NAC and non-NAC cohorts were not performed, limiting our understanding on whether a lower NLR threshold was driven only by NAC administration. However, considering our study and previous reports, it can be assumed that NAC administration lowers the predictive cutoff threshold of NLR. Third, our study assessed the predictive value of the NLR in an NAC-pretreated cohort. Novel therapeutic agents have been proposed for radical treatments, and preliminary results from ongoing trials have highlighted the potential of checkpoint inhibitors [36,37]. Hence, if immunotherapy replaces NAC as the mainstay treatment in the near future, the prognostic value of NLR might be different or become irrelevant. Moreover, given that only 75 patients were confirmed NAC responders, we were not able to perform a reliable analysis of NLR in this group. We believe that predicting survival outcomes in patients with <ypT2N0 disease is crucial, since bladder preservation is advocated in these patients. This is particularly relevant because bladder preservation is usually considered for patients with <ypT2N0 disease, and stratifying this group based on CSS potential using NLR could have significant clinical implications. Lastly, we are aware that other inflammatory ratios have been considered as prognostic markers in oncology. For instance, in penile cancer, the albumin-to-alkaline-phosphatase ratio might predict lymph node involvement [38]. Hence, our results should be compared to other inflammatory markers in the BC setting to decide which ratio has the highest predictive value.

Despite these limitations, we believe that the present study provides a reliable analysis of NLR in RC. One strength of the present study is the novelty of the results, underscoring the prognostic value of post-NAC NLR. To the best of our knowledge, there is limited data on the ability of post-NAC NLR to determine responses and long-term outcomes in urological cancers. Hence, we are aware that further studies should confirm our results before post-NAC NLR can be used clinically as an adjunct to well-established prognostic markers. Nevertheless, its ease and affordability from routine blood tests make it a potentially valuable tool for patient risk stratification.

## 5. Conclusions

In summary, our results emphasise that pre-RC NLR may be a clinically significant predictor of local response and long-term outcomes in patients with MIBC undergoing multidisciplinary treatment. Consequently, we believe that this biomarker may be used as an adjunct when deciding between RC and bladder-preserving therapies after NAC. In addition, NLR may hold promise for patient selection in adjuvant immunotherapy and chemotherapy. Using these findings, future studies should validate our results for real-world applicability, and determine whether an NLR increase during long-term follow-up may precede BC recurrence.

## Figures and Tables

**Table 1:** Baseline patient characteristics.

Variable	NLR ≤ 1.75	NLR > 1.75	*p*-Value
Totals, No.	76	138	
Age, years			0.290
Mean	65.48	66.54	
SD	6.73	7.06	
Sex, No.			0.587
Male	57	108	
Female	19	30	
BMI, No.			0.125
<30 kg/m^2^	58	117	
≥30 kg/m^2^	18	21	
ASA score, No.			0.151
1	3	11	
2	47	75	
3	24	50	
4	1	0	
Smoking status, No.			0.281
Never	18	23	
Former	34	75	
Current	21	38	
Clinical T stage, No.			0.193
cT2	46	67	
cT3	15	30	
cT4	15	41	
Pathological T stage, No.			<0.001
ypT0	27	13	
ypTis/Ta/T1	26	9	
ypT2	5	49	
ypT3	10	30	
ypT4	8	37	
Pathological N stage, No.			0.003
ypN0	65	92	
ypN+	11	46	
Cancer grade, No.			0.567
Low grade	4	5	
High grade	72	133	
Chemotherapy regimen, No.			0.876
ddMVAC	42	82	
Gemcitabine–cisplatin	27	44	
Gemcitabine–carboplatin	2	5	
Gemcitabine–paclitaxel	5	7	

ASA score: American Society of Anesthesiologists score; BMI: body mass index; ddMVAC: dose-dense methotrexate, vinblastine, doxorubicin, and cisplatin; SD standard deviation; NLR: neutrophil-to-lymphocyte ratio.

**Table 2:** Univariate and multivariate analyses for predictors of response (<ypT2N0) to neoadjuvant chemotherapy.

	Univariate				Multivariate			
	OR	95% CI Lower	95% CI Upper	*p*-Value	OR	95% CI Lower	95% CI Upper	*p*-Value
Age	1.007	0.967	1.049	0.730	1.049	0.984	1.119	0.145
Sex								
Male	Ref.	Ref.	Ref.		Ref.	Ref.	Ref.	
Female	1.100	0.567	2.136	0.778	0.683	0.256	1.823	0.446
BMI								
<30 kg/m^2^	Ref.	Ref.	Ref.		Ref.	Ref.	Ref.	
≥30 kg/m^2^	1.559	0.769	3.161	0.218	0.933	0.302	2.879	0.904
ASA score								
1–2	Ref.	Ref.	Ref.		Ref.	Ref.	Ref.	
3–4	0.787	0.429	1.445	0.440	1.000	0.402	2.487	0.999
Smoking status								
Never	Ref.	Ref.	Ref.		Ref.	Ref.	Ref.	
Former	0.613	0.291	1.290	0.197	0.257	0.084	0.790	0.018
Current	0.902	0.400	2.032	0.803	0.537	0.165	1.751	0.302
Clinical T stage								
cT2	Ref.	Ref.	Ref.		Ref.	Ref.	Ref.	
cT3	0.184	0.079	0.431	<0.001	0.064	0.018	0.228	<0.001
cT4	0.102	0.041	0.258	<0.001	0.030	0.008	0.118	<0.001
NLR								
NLR ≤ 1.75	Ref.	Ref.	Ref.		Ref.	Ref.	Ref.	
NLR > 1.75	0.082	0.042	0.161	<0.001	0.045	0.017	0.119	<0.001
Chemotherapy regimen								
ddMVAC	Ref.	Ref.	Ref.		Ref.	Ref.	Ref.	
Gemcitabine–cisplatin	1.024	0.555	1.887	0.940	1.086	0.442	2.672	0.857
Gemcitabine–carboplatin	0.753	0.140	4.047	0.741	0.490	0.048	4.957	0.545
Gemcitabine–paclitaxel	1.346	0.403	4.493	0.629	1.253	0.233	6.749	0.793

ASA score: American Society of Anesthesiologists score; BMI: body mass index; CI: confidence interval; ddMVAC: dose-dense methotrexate, vinblastine, doxorubicin, and cisplatin; OR: odds ratio; NLR: neutrophil-to-lymphocyte ratio; n/a: not applicable; Ref.: Reference category.

**Table 3:** Univariate and multivariate analyses of overall survival and cancer-specific survival.

	Overall Survival				Cancer-Specific Survival			
	HR	95% CI Lower	95% CI Upper	*p*-Value	HR	95% CI Lower	95% CI Upper	*p*-Value
Age	1.009	0.981	1.038	0.534	1.028	0.959	1.103	0.432
Sex								
Male	Ref.	Ref.	Ref.		Ref.	Ref.	Ref.	
Female	1.627	0.985	2.689	0.057	0.391	0.102	1.507	0.173
BMI								
<30 kg/m^2^	Ref.	Ref.	Ref.		Ref.	Ref.	Ref.	
≥30 kg/m^2^	0.958	0.571	1.607	0.871	0.170	0.041	0.701	0.014
ASA score								
1–2	Ref.	Ref.	Ref.		Ref.	Ref.	Ref.	
3–4	1.243	0.807	1.913	0.324	8.161	2.983	22.331	<0.001
Smoking status								
Never	Ref.	Ref.	Ref.		Ref.	Ref.	Ref.	
Former	3.570	1.952	6.530	<0.001	0.788	0.151	4.107	0.777
Current	2.639	1.429	4.871	0.002	0.680	0.104	4.454	0.687
Clinical T stage								
cT2	Ref.	Ref.	Ref.		Ref.	Ref.	Ref.	
cT3	1.874	1.146	3.064	0.012	8.161	2.983	22.331	<0.001
cT4	2.737	1.697	4.413	<0.001	5.522	1.985	15.360	0.001
NLR								
NLR ≤ 1.75	Ref.	Ref.	Ref.		Ref.	Ref.	Ref.	
NLR > 1.75	1.686	1.049	2.711	0.031	2.069	0.789	5.427	0.140
Chemotherapy regimen								
ddMVAC	Ref.	Ref.	Ref.		Ref.	Ref.	Ref.	
Gemcitabine–cisplatin	1.347	0.906	2.002	0.141	0.814	0.317	2.086	0.667
Gemcitabine–carboplatin	1.949	0.586	6.481	0.276	0.895	0.106	7.535	0.919
Gemcitabine–paclitaxel	0.579	0.244	1.376	0.216	n/a	n/a	n/a	n/a

ASA score: American Society of Anesthesiologists score; BMI: body mass index; CI: confidence interval; ddMVAC: dose-dense methotrexate, vinblastine, doxorubicin, and cisplatin; HR: hazard ratio; NLR: neutrophil-to-lymphocyte ratio; n/a: not applicable.

## Data Availability

Source data available at https://osf.io/637xt, accessed date: 27 February 2024.

## Supplementary Materials

The following supporting information can be downloaded at: https://osf.io/7nqzv, accessed date: 18 March 2024, Table S1: Univariate and multivariate analyses for predictors of complete response (ypT0N0) to neoadjuvant chemotherapy.
